# Biochemical and Nutritional Evaluation of *Chlorella* and *Auxenochlorella* Biomasses Relevant for Food Application

**DOI:** 10.3389/fnut.2020.565996

**Published:** 2020-09-30

**Authors:** Greta Canelli, Carmen Tarnutzer, Roberta Carpine, Lukas Neutsch, Christoph J. Bolten, Fabiola Dionisi, Alexander Mathys

**Affiliations:** ^1^Laboratory of Sustainable Food Processing, ETH Zurich, Swiss Federal Institute of Technology, Zurich, Switzerland; ^2^Institute of Chemistry and Biotechnology, ZHAW, Zurich University of Applied Sciences, Wädenswil, Switzerland; ^3^Department of Organic Chemistry, University of Zürich, Zurich, Switzerland; ^4^Nestlé Research, Lausanne, Switzerland

**Keywords:** microalgae, *Chlorella*, *Auxenochlorella*, bioaccessibility, omega-3-polyunsaturated fatty acids, protein, digestion

## Abstract

Microalgae are a source of potentially healthy and sustainable nutrients. However, the bioaccessibility of these nutrients remains uncertain. In this study, we analyzed the biomass composition of five commercial *Chlorella* and *Auxenochlorella* strains, and *Chlorella vulgaris* heterotrophically cultivated in our laboratory. Protein accounted for 65 ± 3% (w w^−1^) dry matter (DM) in all biomasses, except for the lab-grown *C. vulgaris* that contained 20% (w w^−1^) DM protein. The fatty acids content was comparable and ranged between 7 and 10% (w w^−1^) DM. Most of the biomasses had a ω6-polyunsaturated fatty acids (PUFAs)/ω3-PUFAs ratio <4, as recommended by nutritional experts. A recently published harmonized protocol for *in vitro* digestion was used to evaluate fatty acids and protein bioaccessibilities. Protein bioaccessibility ranged between 60 and 74% for commercial *Chlorella* and *Auxenochlorella* biomasses and was 43% for the lab-grown *C. vulgaris*. Fatty acids bioaccessibility was <7% in commercial biomasses and 19% in the lab-grown *C. vulgaris*. Taken together, the results show that microalgae are promising sources of bioaccessible protein. The limited fatty acids bioaccessibility indicates the need for alternative upstream and downstream production strategies.

## Introduction

Microalgal biomass is an emerging sustainable source of proteins, fatty acids, carotenoids, and carbohydrates with potential health benefits for humans (1–4). Moreover, microalgae often have essential amino acids composition meeting FAO requirements, and they are frequently on par with other protein sources, such as soybean and egg (5). *Chlorella* spp. together with *Arthrospira* spp. (known as “Spirulina”) account for the largest production volume (6). These two genera are among the few that are allowed to be consumed as whole biomass in Europe according to European Union food regulations (7). However, their biomass composition can vary considerably. Knowledge of the biochemical profile of the biomass is necessary for the selection of appropriate microalgae for specific food applications. Different biomass compositions for the same species, or even strains, are reported in the literature. This variability is partially due to differences in cultivation conditions. Factors that include pH, temperature, salinity, and nutrient availability can affect the biomass composition (2, 8). The variation that has been described might also be attributed to the different analytical methods used (9). When measuring nitrogen to calculate protein content, species-specific nitrogen-to-protein conversion factors should be used, instead of the standard 6.25. Even though conversion factors may vary depending on growth stage and cultivation conditions, a conversion factor of 6.35 was proposed for *Chlorella vulgaris* (10). In view of this inconsistency, there is need for studies comparing biomass profiles using standardized protocols.

From a nutritional perspective, microalgae present interesting profiles rich in several nutritional and health-beneficial components, such as polyunsaturated fatty acids (PUFAs). In particular, ω3-PUFAs such as α-linolenic acid, are essential fatty acids that must be supplied in the diet, as they cannot be synthesized by the human body (11). Several important indices, termed indices of lipid nutritional quality (INQ), need to be considered when evaluating the fatty acids profile of an ingredient. Nutritional experts have recommended a ω6-PUFAs/ω3-PUFAs ratio <4 as desirable (12–14). Previous studies reported that a ω6/ω3 ratio <4 reduced total mortality by 70% in the prevention of cardiovascular diseases, a ratio of 5 was beneficial for asthma, a ratio of 2–3 reduced rheumatoid arthritis inflammation, and a ratio of 2.5 reduced colorectal cancer cell proliferation (15). There is no general indication on the recommended ω6/ω3 ratio provided by the European Food Safety Authority. However, several European countries established their own recommendations (16). The German-Austrian-Swiss recommendations, as well as the Nutritional Recommendations for the French Population, recommend a ω6/ω3 ratio of 5. The Nordic Nutrition Recommendations considers a ω6/ω3 ratio between 3 and 9 to be adequate (16). The consumption of foods rich in ω3-PUFAs in Western countries is limited and often scarce (17). Therefore, microalgae can be a promising alternative/supplementation to oil sources, such as fish and plant-based sources (18).

Nutrients, such as protein and fatty acids, are present in microalgae, which are surrounded by a cell wall. It is hypothesized that microalgae cell wall, being mainly composed by indigestible polysaccharides, cannot be degraded by the digestive enzymes present in the mouth, stomach, and small intestine (19). This would limit the nutrient digestibility and bioaccessibility, which has not been thoroughly investigated for many important compounds found in microalgae. Bioaccessibility is defined as the fraction of a food/component that is released from the food matrix in the gastrointestinal tract that becomes available for absorption (20). Bioaccessibility is often determined by *in vitro* digestion. Even though *in vitro* methods strongly simplify reality, they have some advantages over *in vivo* methods. Generally, *in vitro* methods are more rapid, less expensive, less labor intensive, do not have ethical restrictions, and are very suitable for mechanistic studies and hypothesis building (21). However, dissimilar protocols are often used for the assessment. The use of a common protocol for *in vitro* digestion is essential for data comparison. In this study, we followed the recently published harmonized INFOGEST 2.0 protocol (22).

Limited literature is available on *Chlorella* and *Auxenochlorella* nutrient bioaccessibilities. For commercial *Chlorella* biomasses, an average protein digestibility of 51 ± 9% was reported (4). In a previous work, we studied the bioaccessibility of fatty acids by an infant *in vitro* digestion model in *C. vulgaris*, which was limited to 3% (23). To date, no study on fatty acids bioaccessibility in adults for *Chlorella* or *Auxenochlorella* has been published.

Considering this knowledge gap, this study evaluated the bioaccessibilities of fatty acids and proteins in several *Chlorella* and *Auxenochlorella* biomasses relevant for food applications using a harmonized protocol. In addition, biochemical composition and nutritional parameters were assessed following standardized procedures. Commercially available biomasses were compared to *C. vulgaris* heterotrophically grown in our laboratory.

## Materials and Methods

### Acquisition of Algal Biomass

Five commercially available dried *Chlorella* and *Auxenochlorella* biomasses were purchased: Alver “Golden Chlorella,” Biotona, Piura, Purasana, and Soleil Vie (Table 1).

**Table 1:** Overview of the powdered *Chlorella* and *Auxenochlorella* biomasses.

**Genus**	**Brand**	**Supplier**	**Place and year of purchase**	**Country of origin**	**Expiry date**	**Treatment after harvest according to the supplier**	**Species**
*Chlorella*	Biotona	KeyPharm, Oostkamp, Belgium	Apo24.ch, Switzerland, 2019	China	03.2022	Dehydrated by a superior drying process	*Chlorella pyrenoidosa*
	Piura	Green Origins, Sheffield, Great Britain	Narayana Verlag, Germany, 2019	Asia	09.2021	Cell walls broken by an high-impact jet spray process before drying and milling	*Chlorella spp*.
	Purasana	Purasana NV, Gullegem, Belgium	Apo24.ch, Switzerland, 2019	Mongolia or Hainan	30.11.2021	Cell walls broken by an unknown treatment	*Chlorella vulgaris*
	Soleil Vie	Montasell SA, La Tour-de-Trême, Switzerland	Coop, Switzerland, 2019	China	26.10.2020	n. a.	*Chlorella vulgaris*
*Auxenochlorella*	Alver “Golden Chlorella”	Golden Chlorella SA, Chardonne, Switzerland	Alver.ch, Switzerland, 2018	n. a.	n. a.	n. a.	*Auxenochlorella protothecoides* (24)

For each biomass, genus, brand, supplier, place and date of purchase, country of origin, treatment after harvest, and species are reported.

Additionally, *C. vulgaris* biomass was heterotrophically produced in our laboratory as previously described (23). *C. vulgaris* (CCALA 256) was obtained from the Culture Collection of Autotrophic Organisms in the Czech Republic. Batch cultivation was performed in a 16-L laboratory bioreactor (Bilfinger Industrial Technologies, Salzburg, Austria) with a working volume of 10 L. The temperature was set at 28 °C, the stirring speed was 300 rpm, the dissolved oxygen tension was kept above 75%, and aeration (4 L min^−1^) was achieved with filter-sterilized air. The pH was kept constant at 7 by automatic addition of H_2_SO_4_ (0.5 M) and NaOH (0.5 M). The medium used for growth was modified BBM (nitrate concentration of 1.5 g L^−1^) enriched with 15 g L^−1^ glucose. The culture was harvested after 7 days of growth, frozen, and freeze-dried for further analysis.

### Microalgae Biomass Composition

For each microalgae species, triplicate samples of dried biomass were analyzed to determine moisture, carbohydrate, protein, fatty acids contents, and fatty acids composition. Moisture content was determined by weighing before and after drying 1.3 ± 0.3 g of biomass for 24 h at 80 °C. The average moisture content was used in the calculation of the biochemical composition, and was expressed as percentage of total dry matter (DM). Carbohydrate content was determined by the anthrone method as previously described (25). This method is commonly used for quantitative measurement of total carbohydrates in microalgae because of its high sensitivity and simplicity (26). Protein content was determined by the Dumas method. Approximately 0.5 g of biomass was transferred to ceramic crucibles and total nitrogen content was analyzed using a TruMac CN device (LECO Corporation, St. Joseph, MI, USA). Protein content was estimated from the total nitrogen content, multiplied by an overall conversion factor of 6.35, as previously proposed for *C. vulgaris* (10). Fatty acids profile of the biomass was determined as previously reported (23). In brief, fatty acids in freeze-dried biomass were directly trans-esterified using 1.5 N methanolic hydrochloric acid solution and analyzed by gas chromatography using an instrument equipped with a split-injection port and flame ionization detection (FID) (7890 A; Agilent Technologies, Basel, Switzerland). The following temperature–time program was used: 50 °C (0.2 min), 50–180 °C (120 °C min^−1^), 180–220 °C (6.7 °C min^−1^), and 220–250 °C (30 °C min^−1^) on a 70% cyanopropyl polysilphenylene-siloxane column with a length of 10 m, internal diameter of 0.1 mm, and film of 0.2 μm (BPX70; SGE Analytical Science, Milton Keynes, UK). Peak identification was performed by comparing the retention times with FAME standards (Nu-Chek Prep. Inc., Elysian, USA). The peak areas were quantified with OpenLab CDS VL software (Agilent Technologies, Basel, Switzerland).

### Indexes of Lipid Nutritional Quality (INQ)

The nutritional quality of the lipid fraction was assessed by five separate indexes. These are calculated based on the concentration of saturated fatty acids (SFAs, C12:0, C14:0, C16:0, and C18:0), monounsaturated fatty acids (MUFAs, C15:1-ɷ5, C16:1-ɷ7, C17:1-ɷ7, C18:1-ɷ9), and polyunsaturated fatty acids (PUFAs, C18:2-ɷ6, C18:3-ɷ3) according to:

(1) Atherogenicity index (AI) = [(C12:0 + (4 × C14:0) + C16:0)]/(MUFAs + ɷ6-PUFAs + ɷ3-PUFAs) (27)
(2) Thrombogenicity index (TI) = (C14:0 + C16:0 + C18:0)/[(0.5 × MUFAs) + (0.5 × ɷ6-PUFAs) + (3 × ɷ3-PUFAs) + (ɷ3-PUFAs/ɷ6-PUFAs)] (27)
(3) Hypocholesterolemic/hypercholesterolemic fatty acids ratio (H/H) = (C18:1-ɷ9 + C18:2-ɷ6 + C 18:3-ɷ3)/(C14:0 + C16:0) (28)
(4) P/S = PUFAs/SFAs
(5) ω6/ω3 = ɷ6-PUFAs/ɷ3-PUFAs.

### Determination of Fatty Acids and Protein Bioaccessibilities

**Figure 1:**
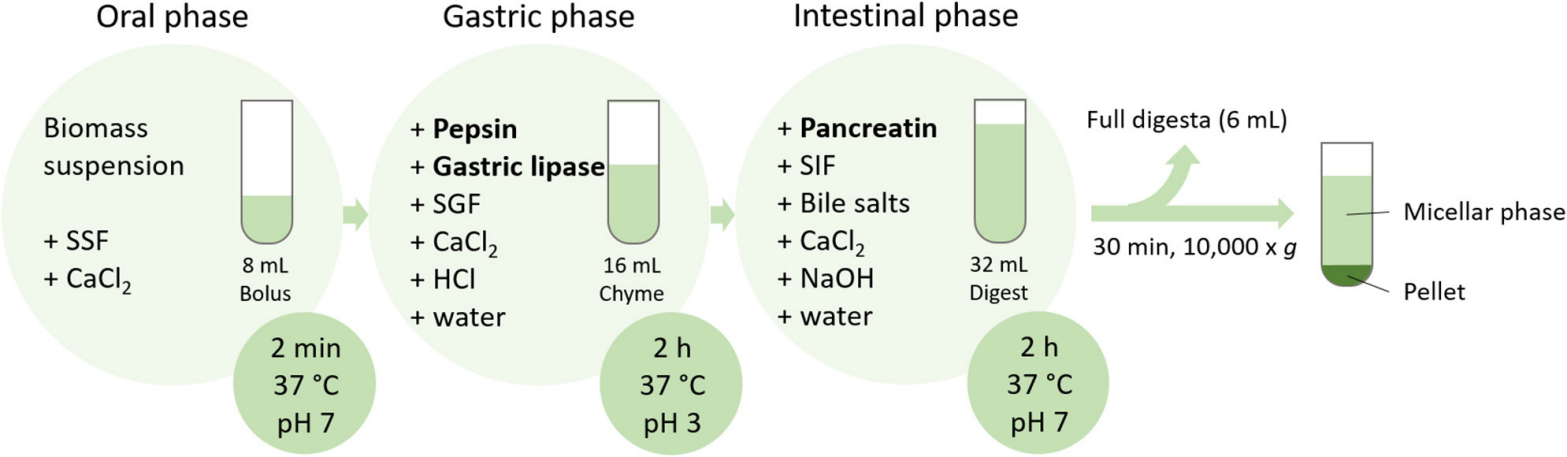
Schematic representation of the *in vitro* digestion protocol applied on biomass suspensions (SSF, simulated salivary fluid; SGF, simulated gastric fluid; SIF, simulated intestinal fluid).

Protein and fatty acids bioaccessibilities were determined by an *in vitro* digestion model (Figure 1), according to the standardized protocol (INFOGEST 2.0) (22). Simulated salivary fluid (SSF), simulated gastric fluid (SGF), and simulated intestinal fluid (SIF) were prepared exactly as recommended by Brodkorb et al. (22). In brief, the digestion was performed in amber glass in a water bath at 37 °C and stirring set at 300 rpm. The oral phase (2 min, pH 7) started with mixing of the biomass (1 g) with water (3.78 mL), SSF (3.2 mL), and CaCl_2_ (20 μL, 0.3 M). To simulate the gastric phase, the oral bolus was then mixed with SGF (6.4 mL) and CaCl_2_ (4 μL, 0.3 M), and the pH adjusted to 3. Pepsin (0.4 mL, 80,000 U mL^−1^; Sigma-Aldrich, Buchs, Switzerland) and gastric lipase (0.4 mL, 2,400 U mL^−1^; Lipolytech, Marseille, France) were added and the total volume was adjusted to 16 mL with water. The pH was constantly adjusted to 3. After 2 h of incubation with stirring, the pH was adjusted to 7 to simulate the intestinal phase and SIF (6.8 mL), CaCl_2_ (32 μL, 0.3 M), pancreatin (4 mL, 800 U mL^−1^; Sigma-Aldrich), and bile salts (2 mL, 0.16 mM; Sigma-Aldrich) were added. Water was added to a total volume of 32 mL. During 2 h incubation with stirring, pH was constantly adjusted to 7. As blank, digestion without biomass was performed. At the end of the intestinal phase, an aliquot (6 mL) of full digesta was snap-frozen with liquid nitrogen and freeze-dried. The residue was centrifuged (30 min, 10,000 × *g*, 4 °C). The micellar phase (supernatant) and the pellet were individually snap-frozen and freeze-dried. As a positive control, infant formula (Aptamil 1; Milupa, Dublin, Ireland) was also subjected to *in vitro* digestion, as complete bioaccessibility was expected for this sample. As a blank, 4 mL of water without microalgal biomass was digested, in order to quantify the fatty acids and nitrogen coming from the enzymes and digestive fluids. This is further referred to as enzyme blank.

Fatty acids and protein contents were measured in the micellar phase, pellet, and full digesta. Total fatty acids were measured as explained in section Microalgae Biomass Composition. Protein content was determined by total nitrogen measurement using a TOC-L equipped with a TN module (Shimadzu Europa, Duisburg, Germany). The dried micellar phase and full digesta (10–20 mg) were dissolved in 15 mL of water and analyzed. Due to poor solubility, the dried pellet was measured using the Dumas method as explained in section Microalgae Biomass Composition. Fatty acids/protein bioaccessibility was defined as the amount of fatty acids/protein incorporated into the micellar phase (corrected by the fatty acids/protein in the micellar phase of enzyme blank) compared the amount of fatty acids/protein in the full digesta (corrected by the fatty acids/protein in the full digesta of enzyme blank), as expressed in percentage (%) in equation 1.

(1) $$\begin{array}{l} Fatty\;acids/protein\;bioaccessibility\;\left(\%\right)=\\ \;\frac{{Fatty\;acids/protein\;}_{micellar\;phase}}{{Fatty\;acids/protein\;}_{full\;digesta}}\;\cdot 100 \end{array}$$

### Data Analysis

Data were shown as the mean ± standard deviation of three independent replicates (*n* = 3). All statistical analyses in the present study were performed using Rstudio software (v4.0.0). The assumptions for parametric tests (equal variance and normality) were tested by Levene's test and Shapiro-Wilk test, respectively, for each factor. When the assumptions were met, one-way ANOVA combined with Tukey's test for multiple comparison was performed to assess statistical significance (*p* < 0.05). For all other factors, a non-parametric tests (Kruskal- Wallis) followed by the Dunn's Multiple Comparison test (*p* < 0.05) was performed. The results of statistical analysis are reported in the Supplementary Material (Table S1).

## Results and Discussion

### Biochemical Characterization of Microalgal Biomasses

Five commercial microalgal biomasses (Biotona, Piura, Purasana, Soleil Vie and Alver) and a biomass of *C. vulgaris* heterotrophically grown in our laboratory (hereafter termed LG-*Chlorella*) were characterized for their macronutrient composition. Results are expressed as percentage on dry matter, as they were corrected for the moisture contents (3.5–5.9%) (Table 2).

**Table 2:** Macronutrient composition (% per 100 g DM) of dried microalgal biomasses.

		**Biotona**	**Piura**	**Purasana**	**Soleil Vie**	**Alver**	**LG-*Chlorella***
	**% dry matter**						
Carbohydrates	EV	11.7 ± 1.7	14.1 ± 0.7	11.0 ± 0.2	9.9 ± 0.4	20.2 ± 0.2	65.0 ± 0.3
	PV	29	17.3	22	5.2	23.5	
Proteins	EV	63.4 ± 0.0	62.7 ± 0.1	65.5 ± 0.1	64.1 ± 0.0	59.6 ± 0.0	18.9 ± 0.0
	PV	58	59.1	60	59.1	63	
Fatty acids	EV	9.8 ± 0.4	9.7 ± 0.6	9.2 ± 1.0	9.0 ± 0.6	10.0 ± 1.2	8.0 ± 0.1
	PV	12	13.4	15	13.4	11	

*Experimental results are expressed as mean ± standard deviation (n = 3) and compared to reference values on the packaging's label. EV, experimental value; PV, packaging value*.

The carbohydrate content in Biotona biomass was 12%, which was lower than the previously reported value of 26% for *C. pyrenoidosa* biomass (1). Purasana and Soleil Vie biomasses (both *C. vulgaris*), contained 10–11% carbohydrate, which was comparable to previously reported values of 10–17% for this species (1, 9). Higher carbohydrate contents were observed for Piura (14%) and Alver (20%), which consist of *Chlorella* spp. and *Auxenochlorella protothecoides* (24), respectively. The yellow color of Alver may suggest that the biomass was grown in heterotrophic conditions (29), which could explain the higher carbohydrate content (30). LG-*Chlorella* that was cultivated under heterotrophic conditions in the presence of glucose had the highest carbohydrate value of 65%.

Protein content was around 60–66% for all biomasses, except LG-*Chlorella*, indicating their potential as protein sources. LG-*Chlorella* contained 20% protein. The difference might be explained by different culture conditions. Nitrogen repletion promotes growth and protein production, whereas nitrogen limitation or depletion retards growth and reduces protein content, but favors starch and/or fat accumulation in cells (23, 31, 32). LG-*Chlorella* was harvested after 4 days of nitrogen limitation, which could explain the high carbohydrate and low protein contents.

The total fatty acids content was similar for all biomasses and ranged between 9 and 10%. These findings are consistent with that of Muys et al. (4). The authors reported an average of 7.5% fatty acids for several commercial *Chlorella* biomasses. In general, *C. vulgaris* can reach fatty acids values between 5 and 40% under optimal growth conditions for cell growth and proliferation and up to 58% under unfavorable conditions (33, 34). High variability in the fatty acids content (6–58%) was also reported for *A. protothecoides*, the species with which Alver was identified (24).

Experimental results were compared with the information on the packaging provided by the supplier. The comparison between package labeling and analysis revealed several differences. The experimental carbohydrate content deviated from what was reported on the labels, with the exception of Alver. This could be explained because carbohydrates on the nutritional label are often calculated by subtracting moisture, protein, fatty acids, and ash content from 100% (35). We observed higher protein and lower fatty acids values for all the biomasses, with the exception of the Alver biomass. Our results confirmed Alver's declaration. Overall, the difference between the supplier's information and experimental results could be explained by the use of different analytical methods. We expressed the fat content as the sum of the total measured fatty acids. Differently, the vendors probably measured the total fat content gravimetrically upon ether extraction, which leads to inclusion of other compounds than fatty acids (36).

#### Fatty Acid Composition

The fatty acid composition was analyzed by identifying the main fatty acids, as well as the proportion of total SFAs, monounsaturated fatty acids (MUFAs), and PUFAs. Ten fatty acids, ranging from of C10:0 to 18:3-ω3, were identified and quantified as the percentage of the total fatty acid content of the algal biomasses (Table 3). The three most abundant fatty acids in all biomasses were palmitic (16:0), linoleic (18:2-ω6), and α-linolenic (18:3-ω3) acids, with values ranging 20–25%, 35–50%, and 10–20%, respectively. Exceptionally, Alver contained a higher amount of oleic acid (40%) than the other biomasses (3–15%), and lower amounts of α-linolenic acid (10%), palmitic acid (16%), and linoleic acid (31%). The residual fatty acids identified (10:0, 14:0, 15:1-ω5, 16:1-ω7, 17:1-ω7, 18:0, and 18:1-ω9) accounted for 10% or less of total fatty acids (with the exception of 18:1-ω9 for LG-*Chlorella*). No EPA and DHA were detected in any biomass. All biomasses had a similar fatty acids profile, except for Alver, which could be attributed to the different genera (24, 29). Compared to other biomasses, Alver displayed lower amounts of SFAs and PUFAs, but higher MUFAs content.

**Table 3:** Fatty acid profile of commercial microalgae biomasses and LG-*Chlorella*.

	**Biotona**	**Piura**	**Purasana**	**Soleil Vie**	**Alver**	**LG-*Chlorella***
10:0	n.d.	n.d.	0.2 ± 0.3	n.d.	n.d.	n.d.
14:0	n.d.	n.d.	0.1 ± 0.2	n.d.	1.8 ± 0.0	n.d.
15:1-ω5	1.3 ± 0.0	1.2 ± 0.0	1.2 ± 0.0	1.0 ± 0.7	n.d.	n.d.
16:0	18.2 ± 0.1	18.7 ± 0.1	18.1 ± 0.3	19.6 ± 1.0	15.9 ± 0.0	20.2 ± 0.0
16:1-ω7	2.6 ± 0.1	1.0 ± 0.0	1.2 ± 0.0	2.7 ± 0.1	n.d.	n.d.
17:1-ω7	7.8 ± 0.1	4.8 ± 0.1	6.4 ± 0.1	8.7 ± 0.4	n.d.	n.d.
18:0	2.3 ± 0.0	2.7 ± 0.0	2.7 ± 0.0	2.7 ± 0.2	1.6 ± 0.0	4.9 ± 0.0
18:1-ω9	2.8 ± 0.0	2.7 ± 0.0	2.1 ± 0.0	2.6 ± 0.1	38.9 ± 0.2	14.8 ± 0.0
18:2-ω6	27.7 ± 0.0	37.5 ± 0.0	34.1 ± 0.5	31.1 ± 1.6	30.6 ± 0.1	40.3 ± 0.1
18:3-ω3	15.1 ± 0.3	7.6 ± 0.1	10.1 ± 0.1	15.8 ± 0.8	9.4 ± 0.1	19.7 ± 0.0
OFAs	22.2 ± 0.1	23.8 ± 0.3	23.9 ± 0.5	15.8 ± 4.6	1.8 ± 0.3	n.d.
Σ SFAs	20.5 ± 0.1	21.4 ± 0.1	21.2 ± 0.7	22.3 ± 1.1	19.3 ± 0.1	25.1 ± 0.0
Σ MUFAs	14.5 ± 0.2	9.7 ± 0.2	10.8 ± 0.2	15.0 ± 1.4	38.9 ± 0.2	14.8 ± 0.0
Σ PUFAs	42.8 ± 0.3	45.1 ± 0.2	44.1 ± 0.6	46.9 ± 2.4	40.0 ± 0.2	60.0 ± 0.1

*Fatty acids are expressed as percentage (%) of the total fatty acids. Results are expressed as mean of triplicates ± standard deviation (n = 3). OFA, Other fatty acid; SFA, Saturated fatty acid; MUFA, Monounsaturated fatty acid; PUFA, Polyunsaturated fatty acid; n.d., not detected*.

Different fatty acid profiles for *C. vulgaris* have been reported (37–39). Lower contents of SFAs were detected in all biomasses (19–25%), compared to the value of 33.5% previously reported by Batista et al. (37). The same study reported a higher proportion of MUFAs (24.9%) instead of the 10–15% in the *Chlorella* biomasses we analyzed. Biomasses of Biotona, Piura, Purasana, Soleil Vie, and Alver had PUFAs contents of 40–47%, which agreed with that detected by Batista et al. (37). LG-*Chlorella* contained 60% PUFAs, indicating its exceptional potential for application in the development of healthy food products.

### Indexes of Lipid Nutritional Quality (INQ)

The nutritional quality of the lipid profiles of the analyzed biomasses was evaluated by five different indexes (Table 4), as previously described (28). A polyunsaturated-to-saturated fatty acids (P/S) ratio <0.45 is considered undesirable in food, because of the potential to induce an increase in blood cholesterol (17). The P/S of the analyzed microalgae biomasses exceeded 2. From a nutritional perspective, a balanced ω6/ω3 fatty acids ratio is important for the prevention and management of obesity, as well as for the reduction of the risk of chronic diseases (13, 15). Typical western diets show preponderance of ω6 over ω3 fatty acids, mainly due to the greater consumption of ω-6 rich vegetable oils (e.g., sunflower, peanut, corn) compared to ω-3 sources, such as fish and nuts (15). All investigated biomasses showed a ω6/ω3 ratio <5. In particular, Biotona, Soleil Vie, and LG-*Chlorella* reported an ideal ratio between 1 and 2.

**Table 4:** Nutritional quality indexes of the lipid fraction of the analyzed microalgal biomasses.

	**Biotona**	**Piura**	**Purasana**	**Soleil Vie**	**Alver**	**LG-*Chlorella***
P/S	2.09 ± 0.01	2.11 ± 0.01	2.08 ± 0.05	2.11 ± 0.01	2.08 ± 0.01	2.39 ± 0.01
ω6/ω3	1.84 ± 0.04	4.93 ± 0.11	3.40 ± 0.06	1.97 ± 0.01	3.27 ± 0.02	2.05 ± 0.00
H/H	2.51 ± 0.01	2.56 ± 0.01	2.53 ± 0.03	2.53 ± 0.02	4.47 ± 0.03	3.70 ± 0.01
AI	0.32 ± 0.00	0.34 ± 0.00	0.34 ± 0.02	0.32 ± 0.00	0.29 ± 0.00	0.27 ± 0.00
TI	0.31 ± 0.00	0.46 ± 0.00	0.40 ± 0.01	0.32 ± 0.00	0.31 ± 0.00	0.29 ± 0.00

*Results are expressed as the mean of triplicates ± standard deviation (n = 3). P/S, polyunsaturated/saturated; ω6/ω3, ɷ6-PUFAs/ɷ3-PUFAs; H/H, hypocholesterolemic/hypercholesterolemic fatty acids ratio; AI, atherogenicity index; TI, thrombogenicity index*.

When studying the functional effect of fatty acids, the hypocholesterolemic fatty acids/hypercholesterolemic fatty acids (H/H) index should be considered. A higher H/H is directly proportional to PUFAs content, and is thought to have beneficial effect on cholesterol level (28). H/H for Biotona, Piura, Purasana, and Soleil Vie were ~2.5, which was higher than the value of 2 reported by Matos et al. (28). Alver and LG-*Chlorella* had even higher H/H values of 4.5 and 3.7, respectively. The H/H values in microalgae were lower compared to that of chia (H/H = 11.4) or flax seeds (H/H = 17.3) (40, 41). When compared to marine fish (H/H = 0.9–2.5), the analyzed microalgal biomass had an excellent H/H ratio (42).

According to Ulbricht and Southgate (27), the atherogenicity index (AI), and thrombogenicity index (TI) evaluate the potential for stimulating platelet aggregation. There are no recommended values for AI and TI. The lower the AI and TI values, the higher the protective potential against heart coronary diseases (40). In addition, recent studies found positive associations between both general and abdominal obesity and AI and TI (43). Furthermore, a positive association between gestational diabetes mellitus and TI was reported in pregnant women (44). Myristic acid (C14:0) and palmitic acid (C16:0) are among the most atherogenic agents, whereas stearic acid (C18:0) is considered thrombogenic but not atherogenic (45). In this study, AI values ranged between 0.27 and 0.34, while TI was 0.29–0.46. The lowest AI and TI of 0.27 and 0.29, respectively, were found in LG-*Chlorella*. These values agreed very well with the data reported for marine fish, where AI = 0.26–0.60 and TI = 0.20–0.44 (42). Chia (AI = 0.09, TI = 0.05) and flax seeds (AI = 0.06, TI = 71.7) presented lower AI and TI, except for a much higher TI in flax seeds (40, 41).

### Fatty Acids and Protein Bioaccessibilities

**Figure 2:**
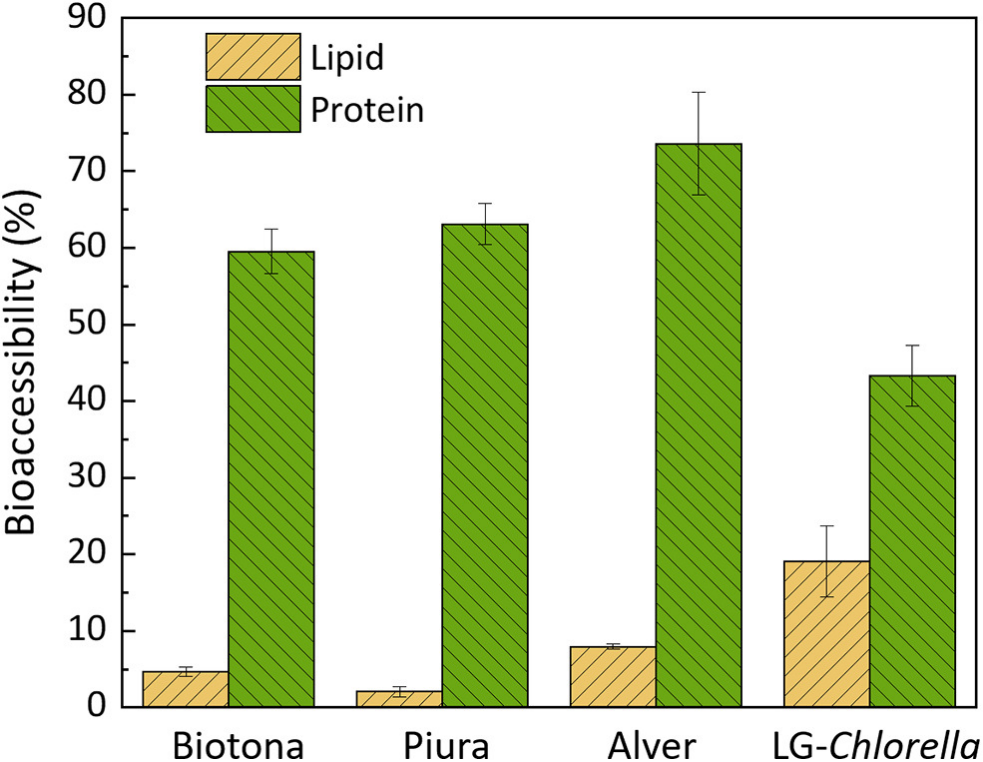
Fatty acids and protein bioaccessibility (%) in microalgae biomasses, expressed as mean of digestion triplicates (*n* = 3) ± standard deviation.

Fatty acids and protein bioaccessibilities in the analyzed biomasses are reported in Figure 2. The validity of the digestion model was tested by including a positive control (infant formula), which showed a protein and fatty acids bioaccessibility of 97.5 ± 3.2% and 88.7 ± 4.5%, respectively. The protein bioaccessibility was 60, 63, 74, and 43% for Biotona, Piura, Alver, and LG-*Chlorella*, respectively. Alver had a higher protein bioaccessibility than Biotona and LG-*Chlorella*. The latter had the lowest protein bioaccessibility of all biomasses. A high polysaccharide content is one of the main factors that negatively influences protein digestibility, as reviewed by Bleakley and Hayes (5). This could explain the lower protein bioaccessibility of LG-*Chlorella*, which had a carbohydrate content of 70%. Piura was reported to have cell walls broken by a high-impact jet spray process before drying and milling. Alver and Biotona biomasses were likely to be disrupted as they had a similar or even a higher protein bioaccessibility than Piura. However, whether cell disruption affected protein bioaccessibility cannot be elucidated from this data, because information about disruption treatment of the biomass from the supplier were unclear.

Overall, the protein bioaccessibility observed in this study is comparable to the 51 ± 9% reported by Muys et al. (4) for commercial *Chlorella* biomasses. The deviation of our results might reflect the different *in vitro* digestion protocol used. We used the latest version of the standardized INFOGEST protocol (22), whereas Muys et al. (4) followed the first version, which did not yet include gastric lipase (21). Although gastric lipase is more relevant for fat digestion, there is always an interplay between any digestive enzyme and nutrient bioaccessibility. Other authors found protein digestibility values between 27 and 70% for *Chlorella*, but followed different protocols (46, 47). The variability found in data reported in literature highlights the importance of using a harmonized protocol.

In case of fatty acids bioaccessibility, LG-*Chlorella* showed the highest value (19%) compared to the commercially available biomasses (<7%). No relevant variation was observed among the commercial biomasses for fatty acids bioaccessibility. Additionally, we calculated the bioaccessibilities of single fatty acids. Results showed that there was no significant difference in the bioaccessibilities between the type of fatty acids (data not shown).

Comparable data for fatty acids bioaccessibility in the literature are scarce. Alternatively, data on bioaccessibility of β-carotene and lutein, which are associated with the fat droplets, are available (34). Gille et al. (48) reported that no β-carotene and only 7% of lutein was bioaccessible in *C. vulgaris* biomass (48). Our results are similar. In addition, they explained the higher bioaccessibility of lutein by its higher hydrophilicity and, therefore, higher release in the aqueous micellar phase compared to β-carotene. Interestingly, protein bioaccessibility values are clearly distinct from the fat bioaccessibility values. Safi et al. (49) reported that *C. vulgaris* proteins are almost all hydro-soluble, which could explain the simpler diffusion of those in the aqueous micellar phase during digestion. Moreover, protein localization within the algae might facilitate their bioaccessibility, with 20% cell wall associated, 30% able to diffuse freely, and 50% located internally (50). It might be that the intracellular and the freely diffusible proteins are more bioaccessible. Contrarily, the very limited fatty acids bioaccessibility may indicate that fatty acids are not able to easily diffuse out of the cell wall, contact digestive enzymes, and become incorporated in the micellar phase (51). In support of our hypothesis, Zhang et al. (52) showed that some of the fatty acids in *Chlorella* are attached to the cell wall, probably linked to carbohydrates by an ether bond. Another reason for the limited fat bioaccessibility could be that free fatty acids that are liberated after hydrolysis have established complexes with proteins/carbohydrates or salts (such as calcium) and precipitated in the pellet obtained by centrifugation after digestion (21).

In general, the incomplete bioaccessibility of fatty acids, and to a lesser extent of proteins, is an indication for the expected poor digestibility of the chitin-like cell wall, for which humans do not possess digestive enzymes.

Further research should explore the nutrient bioaccessibilities of microalgae biomass upon *in vivo* digestion, to overcome the limitations of *in vitro* studies, such as the absence of physiological adaptation in pH and enzyme concentration, interaction with the gut microbiota, and mechanical dynamics (22). Moreover, it would be valuable to investigate nutritional and biochemical qualities of additional microalgae species interesting for the food and nutraceutical industries.

## Conclusions

The study determined the biochemical composition of *Chlorella* and *Auxenochlorella* biomasses. Protein accounted for 65 ± 3% in all biomasses, except for the lab-grown *C. vulgaris* that contained 20% protein. The fatty acids content was comparable and ranged between 7 and 10%. All biomasses showed a relevant fat nutritional quality, with balanced ω6/ω3, P/S, H/H, AI, and TI indexes. The protein bioaccessibility was > 40% for all biomasses, while the fatty acids bioaccessibility was <7% in commercial biomasses and 19% in LG-*Chlorella*. Taken together, the results show that microalgae are promising sources of bioaccessible protein. Regarding fatty acids, their limited bioaccessibility indicates the need for alternative upstream and downstream production strategies.

## Data Availability Statement

The raw data supporting the conclusions of this article will be made available by the authors, without undue reservation.

## Author Contributions

GC and CT: methodology, formal analysis, and writing—original draft. GC and AM: project administration. GC, RC, LN, CB, FD, and AM: conceptualization, writing—review, and editing. AM: funding acquisition. All authors contributed to the article and approved the submitted version.

## Conflict of Interest

CB and FD were employed by the company Nestlé S.A. The remaining authors declare that the research was conducted in the absence of any commercial or financial relationships that could be construed as a potential conflict of interest.
